# Disposable laryngoscope intubation to reduce equipment failure in an emergency out of OR setting - a quality control case study

**DOI:** 10.1186/s12871-022-01956-3

**Published:** 2023-01-10

**Authors:** Colby G. Simmons, Tobias Eckle, Dustin Rogers, Jason D. Williams, Jason C. Brainard

**Affiliations:** 1 Department of Anesthesiology, University of Colorado School of Medicine, 12401 E. 17th Ave Leprino Bldg #734Anschutz Medical Campus, Aurora, CO USA; 2 Department of Biostatistics and Informatics, University of Colorado School of Public Health, Fitzsimons Building, 4th Floor 13001 E. 17th Place Mail Stop B119 Anschutz Medical Campus, Aurora, CO USA; 3 Saint Alphonsus Regional Medical Center, 1055 North Curtis Rd, Boise, ID USA

**Keywords:** Quality Improvement, Airway management, Cost-effectiveness, Environmental sustainability, Patient safety, Value

## Abstract

**Background:**

Reusable laryngoscopes have been reported to be superior to disposable laryngoscopes with plastic blades during emergent intubations. Surprisingly, at our institution a quality reporting system revealed a high number of equipment failures with reusable laryngoscopes in an emergency out-of-OR (operating room) setting. As recent studies indicated an improved quality of disposable laryngoscopes, we hypothesized that a thoroughly evaluated disposable laryngoscope would result in less equipment failure in an emergency out-of-OR setting.

**Methods:**

To perform a more standardized and time efficient analysis, four distinct disposable laryngoscope blade/handle configurations were trialed during standard intubations (*n* = 4 × 30) in the OR by experienced anesthesia providers who completed a 6-question, Likert-scale/open-ended survey for product evaluation. The ‘best’ disposable blade was implemented in an emergency out-of-OR setting and equipment failure rates were monitored over a 3-year period.

**Results:**

Different disposable laryngoscopes were equal regarding sturdiness, illumination and airway visualization. The laryngoscope with the highest overall score was significantly higher scored than the laryngoscope with the lowest overall score. All disposable laryngoscopes were more cost effective than the reusable ones, and the top scored laryngoscope demonstrated the highest 5-year cost-saving ($210 K). Implementation of the top scored disposable laryngoscope into an emergency out-of-OR setting reduced the equipment failure incidence from high 20s to 0.

**Conclusion:**

Disposable laryngoscopes are cost effective and superior to reusable laryngoscopes in an emergency out-of-OR setting. We demonstrate that the implementation of a disposable laryngoscope in the emergency out-of-OR setting resulted in a near elimination of equipment related quality submissions which ultimately enhances patient safety.

## Introduction

Emergency tracheal intubation in an out-of-operating Room (OR) setting can be difficult and has frequently been associated with significant complications [1]. In general, human factors are the most prevalent cause of medical errors during airway management and as such were prominent in the ‘4th National Audit Project of the Royal College of Anaesthetists and Difficult Airway Society’ report in 2011 [2]. Based on a report from 1984, major complications of airway management due to equipment failure are believed to occur in 4% of anesthesia cases, where 10% of all equipment failures are related to the laryngoscope [3]. However, reports on laryngoscope failure in an out-of-OR setting or equipment failure regarding reusable vs disposable laryngoscopes are limited. E.g. one case report exists indicating that during an emergency intubation in the recovery room using a Penlon Crystal laryngoscope with a disposable Macintosh 4 blade, the laryngoscope blade snapped [4].

While reports on equipment failure comparing different types of laryngoscopes is scarce, several studies have compared the performance of reusable and disposable laryngoscopes. In a landmark study on disposable vs reusable laryngoscopes, it was found that single-use metal blades had a significantly decreased rate of failed intubations than reusable metal blades in a rapid sequence induction of anesthesia [5]. In an out-of-hospital study, it was found that the use of plastic disposable laryngoscope blades decreased the success rate of tracheal intubations at the first attempt performed by emergency care providers [6].

Surprisingly, at our institution a review of the hospital safety reporting system showed around 30 complications per year related to airway equipment failure using reusable laryngoscopes with metal blades during out-of-OR emergency intubations. Several patient safety incidents were reported from failed equipment such as unrecognized battery discharge with infrequent usage or damaged handle and laryngoscope blades. Additional review revealed a shortage of reliable traditional laryngoscopes and that shuffling of equipment from one unit to another ultimately resulted in missing equipment. These findings presented a significant patient safety concern.

Recent revised Joint Commission regulations require a more standardized and consistent process for reprocessing and packaging of equipment [7], and a recent report has demonstrated that disposable laryngoscopes are more cost effective than reusable ones [8]. Moreover more recent literature indicated, that disposable laryngoscopes could be at least equally efficient than reusable ones in an emergency out of hospital setting [9]. Thus, a switch from a reusable to a disposable laryngoscope seemed to be the obvious choice to potentially eliminate our observed equipment failures and further improve quality of emergent initiations.

However, no reports exist analyzing the equipment failure rate of reusable vs disposable laryngoscopes in an emergency out-of-OR setting. We hypothesized that a thoroughly evaluated disposable laryngoscope would result in less equipment failure in an emergency out-of-OR setting than the current standard reusable laryngoscope at our institution. Thus, we decided to evaluate four disposable laryngoscopes, implement the highest scored laryngoscope in an emergency out-of-OR setting and monitor equipment failure rates over a 3-year period.

## Methods

### Study design

This Quality Control Case Study was approved by the Institutional Review Board (Colorado Multiple Institutional Review Board [COMIRB]) at the University of Colorado Denver, USA, as exempt and quality improvement project. In addition, the *SQUIRE 2.0* reporting guidelines [10] were applied.

For clinical evaluation, we identified four leading disposable laryngoscope products previously approved within our health system (UCHealth, University of Colorado Health): Teleflex Rusch® TruLite Secure™ blade/handle combination (TLS); Teleflex Rusch® DispoLED™ handle with Green Rusch Lite™ blade (GDL); Flexicare BriteBlade Pro™ blade with BritePro™ Solo handle (BBP); Karl Storz Laryngobloc™ (SLB) - a one-piece laryngoscope with reusable battery pack. The study was conducted at the Anschutz Inpatient Pavilion, which is a 2 tower, 683-bed, tertiary care hospital that serves as the major teaching hospital of the University of Colorado.

The manufacturers provided the equipment for evaluation. Only Macintosh three blades were used for standardization. Thirty intubation trials of each product were conducted (*n* = 120). Laryngoscopes were trialed in the OR setting by non-student, experienced airway management experts (faculty and senior resident Anesthesiologists, Certified-Anesthesiologist Assistants, Certified Registered Nurse Anesthetists). Immediately following direct laryngoscopy and intubation, staff completed a six-question Likert-scale and open-ended survey, based on laryngoscope characteristics previously evaluated in the literature [8]. Five evaluation categories were selected after an extensive literature review and tailored specifically to address the use in an emergent intubation situation: 1) packaging & assembly, 2) illumination & airway visualization, 3) comfort & ease-of-use, 4) sturdiness & rigidity, and 5) a global overall rating. To further improve standardization, attempts were made to have the same staff member trial all four laryngoscopes, ideally during the same day as operating room workflow allowed. Trial participation was at the discretion of the in-room anesthesia team.

### Statistical analysis

Survey results were analyzed using GraphPad Prism 7.00. Given the non-normality and ordinal nature of the Likert scale used for responses, a Kruskal-Walli’s test with *post-hoc* Dunn’s test was used to assess differences among the four laryngoscopes. Significance was assessed at a nominal type I error rate of 5% for each category’s Kruskal-Walli’s test. A subsequent Dunn’s test was used to determine specific pair-wise differences between each of the laryngoscopes with the same type I error rate of 5%. Bonferroni correction to the Dunn’s test was performed to account for multiple comparisons.

### Cost analysis

For economic evaluation, 5-year cost projections were modeled using Microsoft Excel®. Economic projections were based on historical and projected data from our institution. Key drivers of the economic model included: procedural volume and growth rates, useful life of reusable equipment, cost of durable equipment, cost of reusable equipment, lost and damaged inventory, number of handles and blades used per intubation, labor cost, and equipment reprocessing costs (Rüsch® Green Spec® Reusable Laryngoscope Handles and Blades). Modeling was further supplemented with information from additional sources including manufacturer’s published useful life for reusable equipment and Joint Commission guidance. A “base-case” model (assuming 100% reusable equipment; incorporating the current need for replacement of failing inventory as well as repurchase of projected lost inventory) was compared to four alternative economic models (assuming 100% disposable use; one for each disposable model evaluated in the clinical analysis).

### Run chart

Finally, our institutions’ quality reporting system was leveraged to capture any post-implementation effects on a quarterly basis. Conventional run-chart methodology was employed to identify non-random variation as well as shifts and trends in airway equipment quality reporting All reports categorized as either airway equipment “malfunction” or “not available” were included for analysis.

## Results

**Fig. 1:**
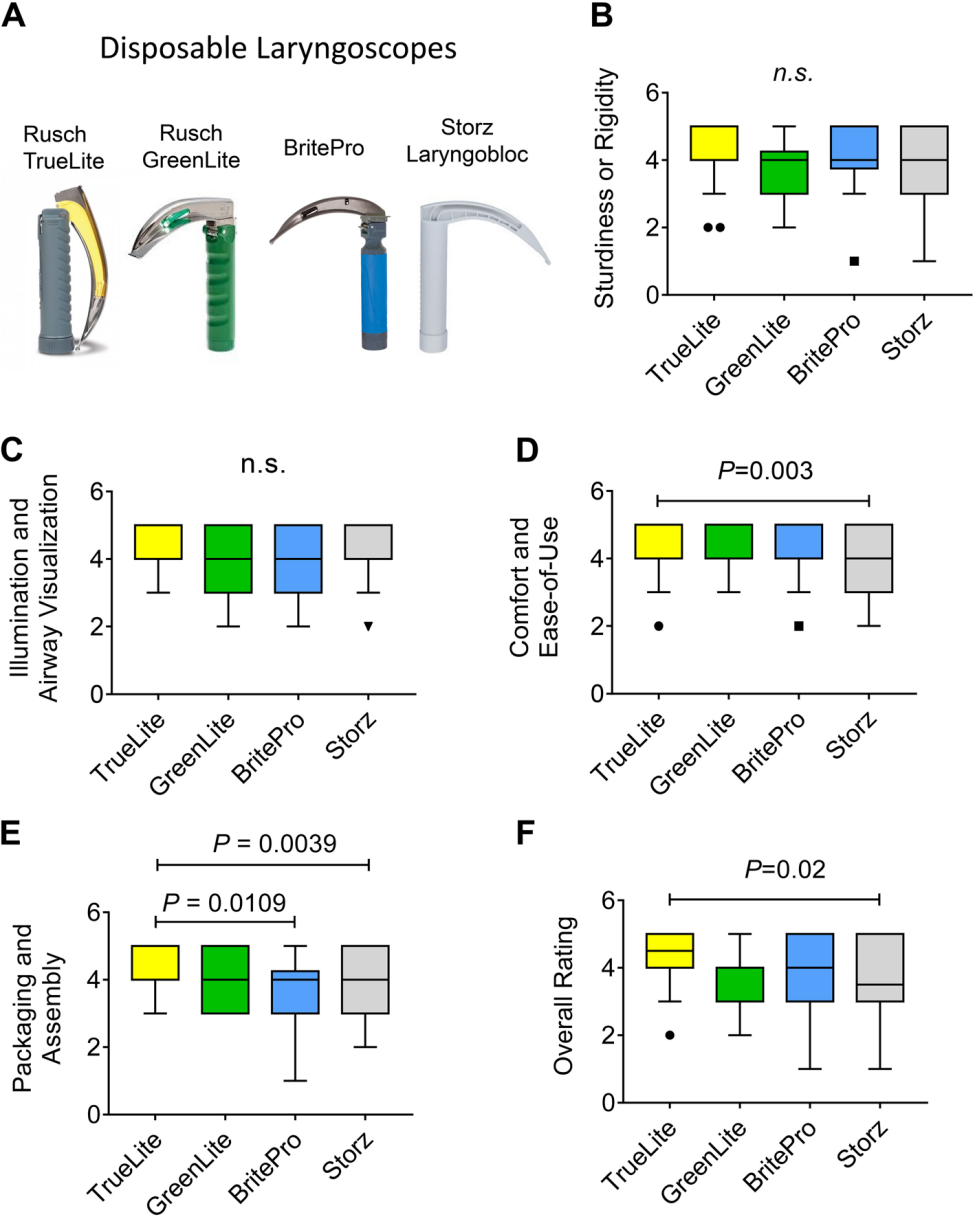
**Quantitative data analysis from a six-question Likert-scale comparing four different disposable laryngoscopes.** (**A**) Blade configurations (**B-F**) Statistical analysis of the six-question Likert-scale for the categories ‘sturdiness & rigidity’, ‘illumination & airway visualization’, ‘comfort & ease-of-use’, ‘packaging & assembly’, and ‘global overall rating’. *N* = 30 per laryngoscope, *P* = 0.05, Kruskal-Walli’s test

Based on approved disposable laryngoscope blades within our health care system (UCHealth), we evaluated the Teleflex Rusch® TruLite Secure™ blade/handle combination (TLS), the Teleflex Rusch® DispoLED™ handle with Green Rusch Lite™ blade (GDL), the Flexicare BriteBlade Pro™ blade with BritePro™ Solo handle (BBP) and the Karl Storz Laryngobloc™ (SLB) in the operating room. The blade configurations are shown in Fig. 1A.

Comparing the TLS, the GDL, the BBP and the SLB model in the category’s ‘sturdiness, rigidity’, ‘illumination and airway visualization’ revealed no significant differences regarding average scoring (Fig. 1B, C). The TLS model was significantly higher scored than the SLB model in the category ‘comfort and ease of use’, (Fig. 1D). Comparing the four models regarding packaging and assembly, the TLS model was significantly higher scored than the BBP or the SLB model **(**Fig. 1E**)**. Finally, the TLS model with the highest overall rating (4.4) demonstrated significantly higher overall scores than the lowest rated SLB model (3.6), as shown in Fig. 1F.

**Fig. 2:**
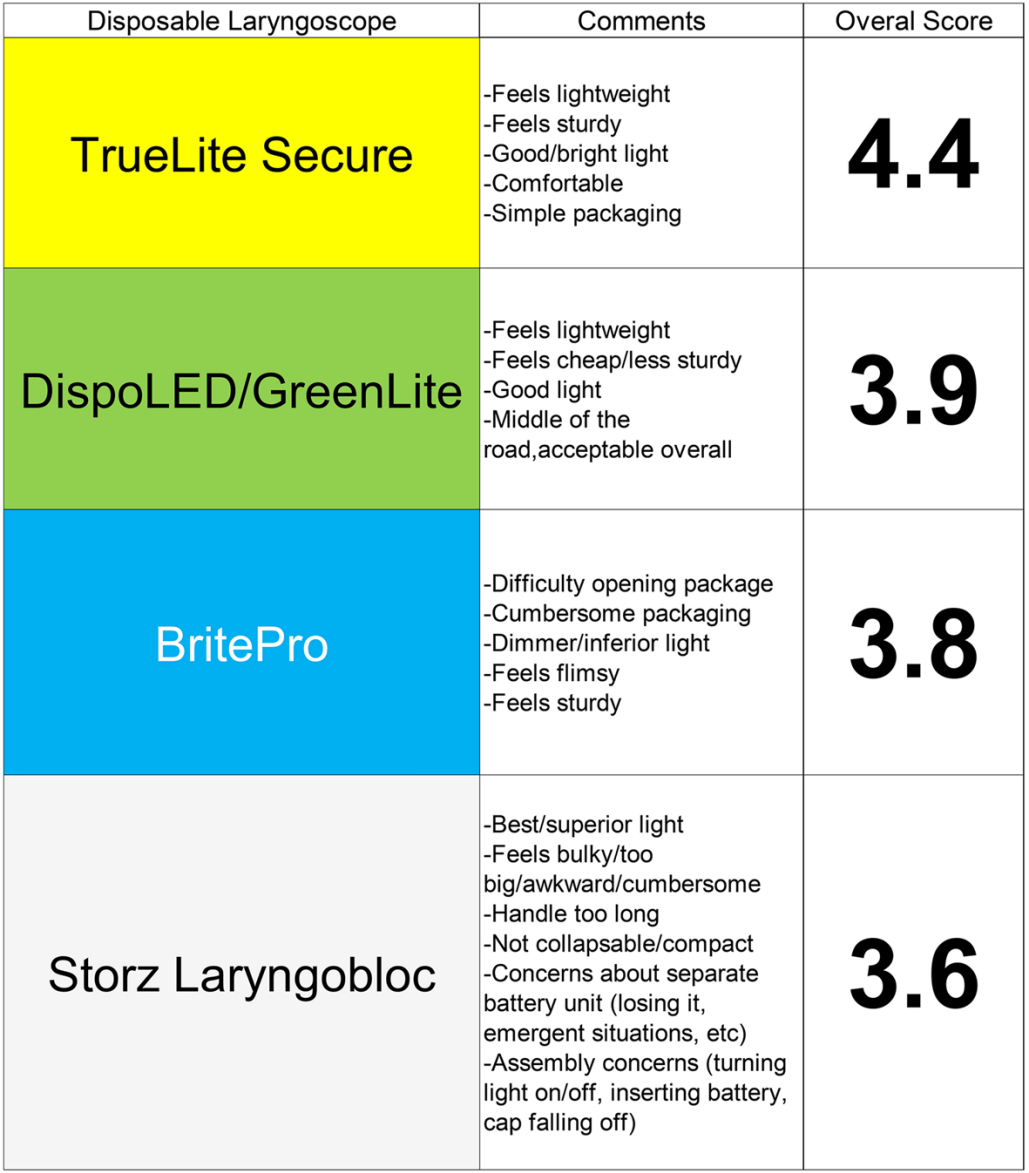
Summary of survey comments and overall scores

Fig. 2 shows details on the qualitative feedback regarding the four laryngoscopes. As can be seen in the comment section, multiple staff provided feedback that the packaging of the second lowest overall scored BBP laryngoscope (3.8) was more difficult to open, specifically commenting on how this could be problematic in an emergency encounter. Similarly, various feedback on the lowest scored SLB laryngoscope (3.6) revealed that the required assembly (insertion of a separate battery pack) would be cumbersome and a distinct disadvantage in an emergent setting compared to the other “all-in-one-and ready-to-use” models. Interestingly, multiple providers commented on the excellent brightness of the SLB model, which however did not have a strong impact on the overall quantitative rating. The second best overall scored GDL laryngoscope (3.9) was overall acceptable but was reported to ‘feel cheaper’ than the overall top scored TLS model. In general, the feedback received was of high quality, very detailed and focused on finding a high-quality disposable laryngoscope for emergent intubations while addressing concerns regarding the use of disposable products. As expected, the top scored TLC model (4.4) had the most positive feedback regarding all 4 evaluated categories.

**Fig. 3:**
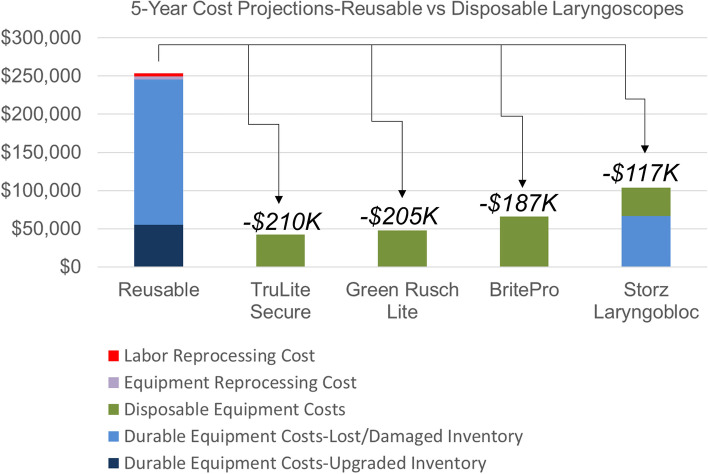
5-year cost projections comparing reusable and disposable laryngoscopes

For the economic evaluation (Fig. 3), all disposable laryngoscope models demonstrated lower 5-year cost-projections compared to the reusable ones at our institution. The models with the highest and lowest cumulative cost projections were the SLB and the TLS, respectively. 5-year cost savings with disposable models ranged from $117,000-$210,000. The SLB model had a uniquely higher cost structure attributable to the required outlay for the reusable battery component. In the base-case scenario, the largest cost component was associated with replacing the projected lost/damaged inventory while the smallest cost component was reprocessing costs, including labor.

**Fig. 4:**
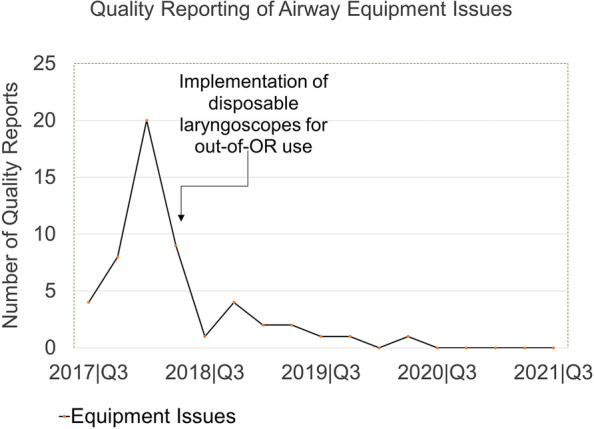
**Run chart for shifts and trends in airway equipment quality reporting.** All reports categorized as either airway equipment “malfunction” or “not available” were included for analysis

Based on the results from our survey and cost analysis, we decided to proceed with the TLS model. The hospital quality reporting system had changed in 2017 which unfortunately resulted in the loss of all reporting data prior to Quarter (Q) 3 in 2017. As shown in Fig. 4 the number of equipment issues were 4 in Q3 of 2017, 8 in Q4 of 2017, 20 in Q1 of 2018 and 9 in Q2 of 2018. The high number of 20 equipment issues in Q1 of 2018 was flagged by the hospital reporting system. Immediately the quality improvement project was initiated and completed within a 2-months period. After implementation of the TLS model in Q2 of 2018 the number of equipment issues dropped to 1 in Q3 of 2018. Thereafter the number of equipment issues remained between 1 and 0 for 9 consecutive quarters. In fact, a comprehensive analysis of the quality reporting data revealed an average of 10.25 equipment issued pre implementation (4 quarters) and an average of 0.9 equipment issues post implementation (13 quarters). Further analysis revealed that implementation of the TLS model resulted in a reduction for both malfunctioning and missing airway equipment. As the reduction of equipment failure by using the new TLS model in an emergency out-of-OR setting was sustained at three years post-intervention, our findings are supportive of our initial hypothesis.

## Discussion

In the current study, we evaluated four disposable laryngoscopes for their implementation into an emergency out-of-OR setting to reduce equipment failure. Here, we found that disposable laryngoscopes were significantly more cost-effective than reusable ones at our institution. We further observed that there were no major equipment failures or disadvantages using disposable laryngoscopes during 120 intubations. We finally demonstrated that implementation of the top scored disposable laryngoscope sustained a zero-failure-rate for the equipment after a 3-year post intervention period.

Studies comparing equipment failure rates of reusable and disposable laryngoscopes in emergency settings are not existent to our knowledge. Interestingly, early studies comparing disposable and reusable laryngoscopes in an emergency out of OR scenario have also not indicated any equipment failure issues but reported lower first attempt intubation rates using disposable laryngoscopes [6]. While it seems reasonable that the sturdy reusable laryngoscopes would deliver better first attempt intubation results, more recent evaluations of this matter did not find any differences between reusable and disposable laryngoscopes [9]. This most up to date observation is most likely due to an improved manufacturing quality of disposable laryngoscopes. In fact, in the current study we also did not observe any equipment failures during 120 intubations using 4 different reusable laryngoscopes.

For the current study we chose the operating room and experienced anesthesiologists to evaluate the disposable blades. The decision was mainly driven by the fact that it would be 1) more time efficient and 2) most likely result in higher quality data with better prediction of the outcome. Indeed, we could have missed certain equipment issues which only would have become apparent in an emergency setting; however, the outcome of our study fully supports our initial decision making. Regardless, we cannot say for sure if any of the four disposable laryngoscopes would have delivered the same results. Future, more comprehensive studies comparing different disposable laryngoscopes will therefore be necessary to fully understand their value in an emergency out-of-OR setting.

Based on published [8] and online available evidence (https://anesthesiaexperts.com), disposable laryngoscopes are more cost efficient than reusable ones. Indeed, the predicted 5-year-cost savings with our disposable laryngoscope was significant. Considering current Joint Commission regulations in concert with lowers costs and potentially no equipment failure rates, the disposable laryngoscope seems the obvious choice for any situation. However, studies have shown that disposable laryngoscopes result in up to 25 more greenhouse gas emissions using standard U.S. energy mix, when compared reusable laryngoscopes [11]. Even more concerning, pollution is a leading cause of non-communicable disease, responsible for an annual 9 million deaths, or 16% of annual deaths globally [12]. As such, current efforts are undertaken to find a middle ground between the use of disposable and reusable equipment to keep health care sustainable [13]. There are also multiple small studies demonstrating that the single use blade has an inferior performance owing to the higher deformability of the blade/joint, especially those comprised of plastic materials. Increased deformability makes vocal cord visualization more difficult [11]. Moreover, it further has been reported that reusable handle/blades would be more economical than single use handle/blades if reusable handles last through 4-5 uses, and reusable blades through 5-7 uses, before loss [11]. Considering these concerns, the use of reusable laryngoscopes should therefore probably only be implemented in an emergency out-of-OR setting with significant lower intubation rates than the busy operating room.

The authors recognize that several limitations exist regarding the study and the findings. As the study was performed at one academic institution, the findings cannot simply be adopted by other institutions. E.g., regarding the cost analysis, we had a relative high cost for lost and damaged equipment regarding the reusable laryngoscopes. While not uncommon for an emergency out-of-OR situation, it might not be the case elsewhere. Further, the standard reusable laryngoscope used at our institution might have a higher incidence of equipment issues in general when compared to other reusable handle/blade configurations. Also, the pre-implementation period was quite short and shorter than the post implementation period. As such the causal correlation between implementation and equipment failure reduction is weakened. In addition, given the unavailability of data before Q3 2017 and the subsequent short pre implementation period, the observed course might represent a regression to the mean of an outlier.

In summary, our study indicates that disposable laryngoscopes have no major failure rates, which suggests advancement in the quality of disposable laryngoscopes and might support recommending disposable laryngoscopes for the use in emergency intubation encounters [14]. Based on our study results, the TLS model was proposed and endorsed by our hospital administration. Rollout of the TLS model in the emergency department, intensive care units, emergency airway trays and code carts across the hospital improved efficiency and efficacy of patient care, resolved patient safety concerns, and continued to generate a significant cost saving for the health care system. Post-rollout surveillance demonstrated continued staff satisfaction and no critical failures per our quality reporting system. The post-intervention run chart illustrates a sustained elimination of airway equipment issues. In fact, zero reports of malfunctioning airway in the final 3 years of post-intervention monitoring, underscores the durability and safety of disposable laryngoscopes for emergency out-of-OR use at our institution.

## Data Availability

All data are available upon request. Any request for additional information should be directed to the corresponding author.
